# Discriminatory validity of the Aspects of Wheelchair Mobility Test as demonstrated by a comparison of four wheelchair types designed for use in low-resource areas

**DOI:** 10.4102/ajod.v6i0.332

**Published:** 2017-09-08

**Authors:** Karen L. Rispin, Elisa Hamm, Joy Wee

**Affiliations:** 1 Department of Biology, Tourneau University, United States; 2 Kinesiology Department, LeTourneau University, United States; 3 School of Rehabilitation Therapy, Queens University, Canada

## Abstract

**Background:**

Comparative effectiveness research on wheelchairs available in low-resource areas is needed to enable effective use of limited funds. Mobility on commonly encountered rolling environments is a key aspect of function. High variation in capacity among wheelchair users can mask changes in mobility because of wheelchair design. A repeated measures protocol in which the participants use one type of wheelchair and then another minimises the impact of individual variation.

**Objectives:**

The Aspects of Wheelchair Mobility Test (AWMT) was designed to be used in repeated measures studies in low-resource areas. It measures the impact of different wheelchair types on physical performance in commonly encountered rolling environments and provides an opportunity for qualitative and quantitative participant response. This study sought to confirm the ability of the AWMT to discern differences in mobility because of wheelchair design.

**Method:**

Participants were wheelchair users at a boarding school for students with disabilities in a low-resource area. Each participant completed timed tests on measured tracks on rough and smooth surfaces, in tight spaces and over curbs. Four types of wheelchairs designed for use in low-resource areas were included.

**Results:**

The protocol demonstrated the ability to discriminate changes in mobility of individuals because of wheelchair type.

**Conclusion:**

Comparative effectiveness studies with this protocol can enable beneficial change. This is illustrated by design alterations by wheelchair manufacturers in response to results.

## Introduction

Research directly comparing different health-related interventions is essential to confirm that the goals of those interventions are achieved (Horn & Gassaway 2007; Jutai et al. 2005). Comparative effectiveness research for wheelchairs intended for use in resource-limited environments is needed (Borg & Khasnabis 2008; WHO 2011).

The ability to move over different surfaces encountered in daily routine is a key aspect of wheelchair effectiveness (Kirby 2011; Mortenson, Miller & Auger 2008). Wheelchair users and family members have often placed highest priority on mobility and the psychosocial benefits that result from it (Hosseini et al. 2012). In fact, wheelchairs are provided specifically to those people who are unable to get around in their daily environment effectively by walking. Therefore, comparative effectiveness studies on the mobility facilitated by different wheelchair types on commonly encountered rolling environments are needed (American Medical Association 1994; Borg & Khasnabis 2008; Matter et al. 2016).

Wheelchairs intended for use in low-resource areas should enable good mobility on commonly encountered rolling surfaces. Wheelchairs designed for use in the United States and Europe may not provide adequate mobility because of the differences in the environments of daily life. In developed areas, populations spend more time indoors on smooth surfaces than they typically do in low-resource areas; therefore there is more need for rough terrain wheelchairs for low-resource areas (Winter et al. 2010). Ramps and wheelchair friendly public transportation are also more likely to be available in wealthier regions (Borg, Lindström & Larsson 2011b). In contrast, wheelchair users in low-resource areas often spend considerable time outdoors where they and their wheelchairs encounter rough terrain, and where public transportation is difficult (Blanford et al. 2012; Borg et al. 2011b; Monk & Wee 2008; Sietchiping, Permezel & Ngomsi 2012). In low-resource areas, living spaces such as houses and schools may be small and sometimes crowded; therefore, the ability to move a wheelchair through tight spaces is also crucial; building codes may not require ramps and other modifications that make wheelchair access easy; thus, coping with curbs is necessary (Borg et al. 2011b; Matter et al. 2016). The environment is so different at some locations that donated wheelchairs from more developed settings have been found to be nearly useless (Mukherjee & Samanta 2005). Wheelchair centre of gravity, caster and wheel sizes, wheelbase length and other design characteristics may impact the ease of movement in different rolling environments quite differently (Cowan et al. 2009). Characteristics of casters and tires including the quality, texture, firmness and condition of the bearings and wheels also impact ease of rolling (Frank & Abel 1989; Kauzlarich & Thacker 1985; VanderWiel et al. 2016).

Organisations manufacturing and providing wheelchairs for low-resource areas face tight financial constraints that are addressed by different organisations in different ways (USAID/WHO 2012). Often, wheelchair availability in low-resource areas may limit the ability of therapists to ideally fit wheelchair users. For example, active users may be put into a range of more or less ideally appropriate wheelchairs depending on what is available (Gartz et al. 2016). The type of wheelchair provided often simply depends on which type is available when one is needed. Studies carried out in the environments where the wheelchairs are used are essential to provide feedback to maximise effective use of limited funds (Jutai et al. 2005).

One of the challenges of comparative studies is the great variation in capability among wheelchair users (Hoenig, Giacobbi & Levy 2007; May 1997; Mortenson et al. 2008). Variations among wheelchair users in skill and capability level can mask the negative impact of a poor wheelchair design. Unless a protocol is used in a repeated measures study design in which the user completes a test in one wheelchair and then another, the impact of wheelchair design on mobility may not be apparent (Hoenig et al. 2015; Mortenson et al. 2008; Rispin & Wee 2015). This type of repeated measures study protocol minimises the effect of individual variation because a participant is compared only with himself or herself (Coutinho, Neto & Beraldo 2014; Neto, Coutinho & Beraldo 2014; Walpole et al. 2006).

Even in a repeated measures protocol, a strongly skilled wheelchair user may be able to roll in most environments in almost any wheelchair; therefore, the ability to discern the effect of different wheelchair designs requires high discriminatory validity. Study design impacts discriminatory validity in several ways. Objectively measured data such as velocity may be considerably lower in one chair type than another when rolling on rough ground, but a study design which only uses a limited categorical response or completion score would indicate the task was completed successfully in both chairs. Objectively measured physical performance data such as velocity and heart rate also minimise variation because of raters’ perceptive frames (May 1997). In addition, unlike categorical data, objectively measured data is often suitable for powerful parametric statistical tools such as analysis of variance (ANOVA) (May 1997; Walpole et al. 2006). The length of a timed test impacts discriminatory power with longer tests amplifying differences in velocity (Kosak & Smith 2005). Longer timed roll tests are also more likely than shorter tests to discern differences. A change in the ease of rolling which impacts energy cost is more evident as participants move into aerobic exercise about 2 min into a test (Berne et al. 2004; Kosak & Smith 2005).

Direct questionnaire feedback from wheelchair users provides insights on capability and mobility not available any other way (Neale & Strang 2015; Reeve et al. 2013). Questionnaire design can enhance or reduce the ability to discriminate differences (May 1997; Reips & Funke 2008). Visual analogue scale (VAS) question format produces continuous data which has been considered suitable for parametric statistical analysis (Philip 1990; Walpole et al. 2006). Qualitative comments directly provide the wheelchair user’s understanding of the reasons for ease or difficulty (Neale & Strang 2015). Discriminatory validity is enhanced by a mixed methods protocol which includes qualitative data, allowing wider scope of understanding and triangulation (Fielding 2012). The Quebec User Evaluation of Satisfaction with Assistive Technology obtains user feedback on their satisfaction with any assistive device (Demers et al. 2002). It can and has been used in comparative effectiveness studies assessing user satisfaction with assistive technology (Deems-Dluhy et al. 2016; Sadiya, Pattnaik & Mohanty 2016). However, it is not a physical performance measure and does not supply information specific to wheelchair mobility.

Questionnaires aimed at assessing individual capability and physical performance include Functional Independence Measure and the Barthel Index (Kumar et al. 2013; Ottenbacher et al. 1996; Wade & Collin 1988). Wheelchair-specific questionnaires include the Wheelchair Skills Test Questionnaire and Functioning Every Day in a Wheelchair questionnaire (Kirby et al. 2004; Mills et al. 2002).

Directly measured or observed physical performance measures intended to assess a wheelchair user’s capabilities are often called skills tests (Kirby 2011; Oyster et al. 2012). They include the Wheelchair Skills Test, the Wheelchair Propulsion Test, the Wheelchair Users Functional Assessment, the Wheelchair Circuit, the Obstacle Course Assessment of Wheelchair User Performance and the Wheelchair Physical Functional Performance (Askari et al. 2013; Cress et al. 2002; Fliess-Douer et al. 2010; Kirby et al. 2004; Mortenson, Miller & Miller-Pogar 2007; Routhier et al. 2004; Rushton et al. 2013; Stanley et al. 2003). The above measures are not primarily designed for use in repeated measures studies assessing differences in mobility because of wheelchair design. In spite of its focus on the evaluation of individual wheelchair user’s skills and capacity, the Wheelchair Skills Test has been used in studies comparing design changes in tilt in space and anti-tip devices. In the tilt in space study, the Quebec User Evaluation of Satisfaction with Assistive Technology and a VAS question regarding perceived exertion were also completed (Kirby et al. 2008a, 2008b).

Objective quantitative data on mobility have also been obtained in laboratory settings (Askari et al. 2013; Coutinho et al. 2014; Cowan et al. 2008; Cress et al. 2002; Neto et al. 2014; Yang et al. 2006). However, a laboratory setting does not perfectly mimic the conditions of daily use. It is important that effectiveness studies are also done in the environment where wheelchairs are used (Jutai et al. 2005; Toro et al. 2012; WHO 2015).

The Aspects of Wheelchair Mobility Protocol (AWMP) was developed to be used in low-resource areas in a repeated measures format to discern differences in mobility because of wheelchair design (Rispin & Wee 2015). There is a tension between every rolling environment that may be of interest, and keeping a protocol short, simple and usable. Because smooth and rough surfaces, tight spaces and low curbs are commonly encountered, and because each interacts somewhat differently with the wheelchair design, these surfaces were included in the AWMP (Rispin & Wee 2015). Face validity is the logical subjective expectation that a protocol will test its target construct (Jerosch-Herold 2005). To that end, mobility was directly measured using timed roll tests similar to the long validated timed walk test protocols (Enright 2003; Rispin & Wee 2015).

Methods were selected with the intention that quantitative data would be continuous and suitable for powerful parametric statistical tools such as ANOVA (Rispin & Wee 2015; Walpole et al. 2006). Performance tests were of sufficient duration to include the transition to aerobic respiration (Neto et al. 2014; Rispin & Wee 2015). Visual analogue scale responses and comments were solicited from participants, and exercise and resting heart rate were monitored using research grade heart rate monitors (Crapo et al. 2002; Rispin & Wee 2015). The format of the participant response questions in AWMP was based on that used in the Lower Limb Function Questionnaire. This format was selected because VAS format is very likely to provide data suitable for parametric statistical analysis tools, and because each question includes an opportunity to write a comment providing qualitative explanatory information (Funk et al. 2016).

Measurements of heart rate or oxygen consumption provide continuous objective data on the energy cost of movement (Coutinho et al. 2014; Neto et al. 2014). In earlier iterations of the AWMP, the physiological cost index (PCI) was calculated rather than directly comparing exercise heart rate (Rispin & Wee 2015). Slowing down when encountering difficulty or awkwardness is the strategy that has enabled timed walk tests to be validated by measuring difficulty in ambulation (Holland et al. 2014). However, slowing down is not the only response to a greater difficulty in moving. Continuing at the same pace and spending, more energy per unit time is the strategy of dealing with difficulty that underlies monitoring heart rate and oxygen consumption per unit time as a measure of difficulty of movement (Conger & Bassett 2011). PCI calculation includes velocity, and thus includes both strategies of dealing with difficulty; however, this makes it difficult to tell which strategy is most commonly used (Ijzerman & Nene 2002). Circadian rhythm, the cost of digestion, body temperature and other factors have a larger proportional impact on non-exercise heart rate than on exercise heart rate. Because PCI calculation includes non-exercise heart rate, these factors would likely impact PCI more than they would do the direct measurement of exercise heart rate (Berne et al. 2004).

The objective of this study was to obtain data that sheds light on the mobility provided by wheelchairs designed for low-resource areas as they roll on surfaces and in situations commonly encountered there. Our hypothesis was that an updated version of the AWMP which used exercise heart rate instead of PCI would have the discriminatory validity to provide comparative effectiveness data on four types of wheelchairs commonly provided to wheelchair users with strong upper bodies at our study site. Specifically, we hypothesised that ANOVA results for velocity, heart rate and participant response VAS scores would indicate significant differences between wheelchair types. Participant comments would shed light on the perceived reasons for ease or difficulty. Meaningful discriminatory validity would be confirmed by results which would enable wheelchair manufacturers to make responsive changes.

## Methods

### Study site

This study was conducted in partnership with an organisation that provides rehabilitation to students at a boarding school for students with disabilities in Kenya. The location included a primary and secondary school, and participants were drawn from both. Because students with disabilities in Kenya are often not able to attend local schools, some had begun school at an older age. This resulted in a wide age range in study participants. Wheelchair users at the site regularly traverse paved and unpaved areas, curbs and tight spaces.

### Participants

A convenience sample of participants was recruited from wheelchair using students. Local therapists identified students of appropriate size and disability to use the study wheelchairs safely, and who had the ability to self-propel a manual wheelchair without stress on unpaved surfaces. The second criterion was added with the presupposition that strong wheelchair users would provide more complete data sets for all tracks, and would therefore increase the statistical power of ANOVA analysis across tracks and wheelchairs. Participation was voluntary, and participants could withdraw at any time or choose not to complete any task.

### Wheelchairs tested

**FIGURE 1:**
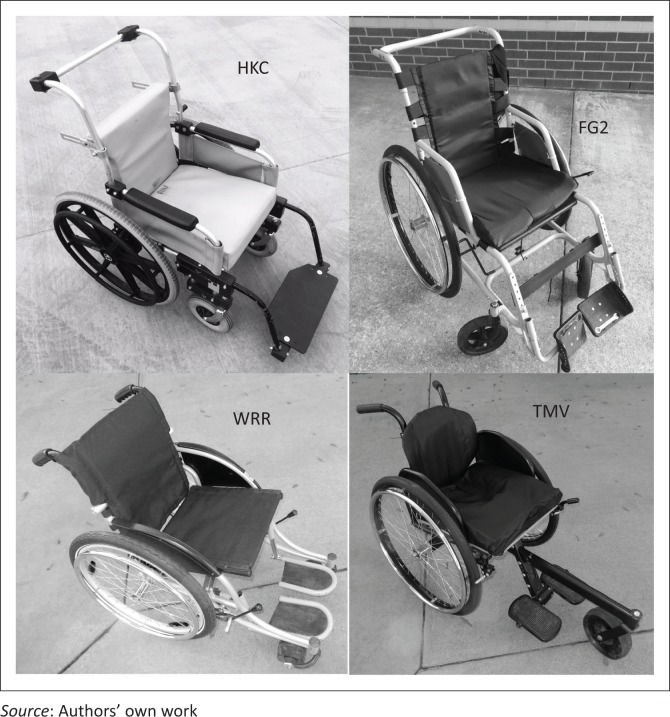
The wheelchairs utilised, shown as they were commonly configured at our location for active users and the way they were configured for this study.

The study utilised four wheelchair types intended for provision in low-resource areas. All four types were commonly provided to wheelchair users with strong upper bodies at our study site, and were available at our study site. They included the Free Wheelchair Mission Generation 2 (FG2) manufactured by Free Wheelchair Mission (Free Wheelchair Mission), the Hope Haven KidChair (HKC) manufactured by Hope Haven (Hope Haven International), the Whirlwind RoughRider (WRR) manufactured by Whirlwind (Whirlwind) and the Motivation Rough Terrain (MRT) wheelchair manufactured by Motivation (Motivation, 2011). Additional information on characteristics of the wheelchair types such as dimensions, weight and so on is available through the manufactures whose websites are cited above. Because AWMP is intended to provide real-world comparative effectiveness data, the wheelchairs were set up in the configuration most commonly used for active wheelchair users at our study site. Photos of the four wheelchairs in the configurations used in this study are given in Figure 1. The width of the seat of the wheelchairs used in this study was the option closest to 33 cm for all chair types. This was chosen because this width was suitable to a large group of wheelchair users at our study site.

This study was part of a long-term study focused on FG2 and HKC wheelchairs. Broader comparison with other wheelchair options for active users at our study site was desired; therefore, MRT and WRR were also included. However, there was no sufficient time to compare all four wheelchairs on all tracks; therefore, only the curb track, the most challenging, was chosen for use with all four wheelchairs.

### Testing protocol

On arriving at the study location, researchers looked for areas appropriate to set up measured tracks incorporating rough, smooth, tight spaces and curbs. For each of the four rolling environments, a looped track was set up and measured using a survey wheel. Rough and smooth tracks were to be of 6 min duration similar to the 6 min timed walk test. Curb and tight tracks were of 3 min duration because of greater difficulty of the curb track and repeated turns on the tight track. Curb and rough rolling environments were included partly to prevent a ceiling effect. HKC and FG2 wheelchairs were used on the tight, rough and smooth surface tracks. All four wheelchair types were used on the curb track to provide a broader comparison for wheelchair function on that track.

**FIGURE 2:**
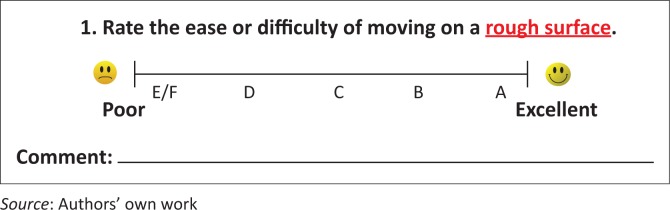
A participant feedback question showing the format used for these questions.

Each participant was asked to attempt to complete each track in each wheelchair utilised on that track. A low discrepancy shifting pattern of wheelchairs and rolling environments was used to avoid skewing of results by the order of testing. If the study wheelchair was not their own wheelchair type, participants were given a few minutes to accommodate to the wheelchair before testing began. To avoid fatigue, data collection for different wheelchairs was done on different days, and participants were pushed between track locations. Distance travelled was measured by counting the number of times the loop was completed, and using a survey wheel to measure the length of the final incomplete loop. Velocity was calculated by dividing distance travelled by test time. After each track, participants were asked to rate the ease or difficulty of movement on a 10-cm VAS and to provide a qualitative explanatory comment (see Figure 2 for question format). Participants wore PolarPro 800 heart rate monitors and watches. Non-exercise heart rate was taken while the participants sat quietly for 5 min before testing began. Each subsequent test was started only when a participant’s heart rate had returned to their initial non-exercise heart rate. Heart rate monitor data were downloaded to a computer, the time period after the first 2 min of each test was selected and the mean heart rate for that time period was calculated.

### Analysis

MiniTab statistical analysis program was utilised for the Anderson–Darling test for normality, and repeated measures ANOVA with Tukey’s simultaneous comparison of means. If a proportion of participants withdrew from a test on a particular track, a chi-squared analysis of proportions test was calculated. Qualitative comments were coded into categories using an open-ended conventional content analysis method in which the content of the comments guided the formation of categories. The number of comments for each category was counted across wheelchair types and tests.

### Ethical consideration

The study protocol was approved by the authors’ universities and by the partner organisations. Participants over 18 years of age provided informed written consent. Those under 18 years of age provided informed written assent and their guardians provided informed written consent.

## Results

### Track characteristics

The rough track was 31.2 m in length on an earth and gravel road around a cul-de-sac with a central circular garden. The smooth track was 60.2 m in length on the paved deck around the school’s outdoor swimming pool. Outside the dining hall, on a cement surface, there was a square area 1.5 m wide, raised 9 cm above the rest of the surface; the curb track was 10 m in length and traversed the raised area twice. For the tight track, four straight-backed school chairs were set in a row 1 m apart on an indoor cement floor. The 12 m track was a figure eight pattern around the middle two of the four chairs. The length of each track was measured using a survey wheel.

### Participants

In total, 30 participants joined the study (age 13.5, SD 3.5, gender 17 male and 13 female). See Table 1 for numbers of participants whose long term wheelchair was one of the types of wheelchairs included in this study, and for diagnoses as provided by our partner organisation.

**TABLE 1a:** Number of participants who were long-term users of a type of wheelchair included in this study, and diagnoses of participants as provided by our partner organisation.

Number of participants	Wheelchair types in long term use
8	Hope Haven KidChair (HKC)
6	Whirlwind RoughRider (WRR)
5	Motivation Rough Terrain (MRT)
2	Free Wheelchair Mission Generation 2 (FG2)
9	Wheelchair types not included in this study

*Source*: Authors’ own work

**TABLE 1b:** Number of participants who were long-term users of a type of wheelchair included in this study, and diagnoses of participants as provided by our partner organisation.

Number of participants	Diagnosis
17	Spinal condition^a^
5	Limb deficiencies^b^
3	Cerebral palsy like neural damage^c^
3	Not provided
2	Muscular dystrophy

*Source*: Authors’ own work

a , includes 12 spina bifida, 3 traumatic spinal cord injury, 1 tuberculosis of the spine, 1 epidermoid cyst.

b , includes 2 congenital deformation, 1 arthrogryposis, 1 osteogenesis imperfecta, 1 bilateral amputation.

c , includes 2 cerebral palsy, 1 malaria in central nervous system.

### Completion rates

All 30 participants completed the two-way comparison on rough, smooth and tight tracks in HKC and FG2 wheelchairs. On the rough track, heart rate and velocity data for two participants were lost when a researcher’s computer crashed. All participants attempted the curb track in all four wheelchair types; however, 18 participants chose not to complete it in HKC, 10 in FG2, 6 in MRT and 5 in WRR. Explanatory comments for those who did not complete indicated difficulty and fatigue.

### Results for two-way comparison

**FIGURE 3:**
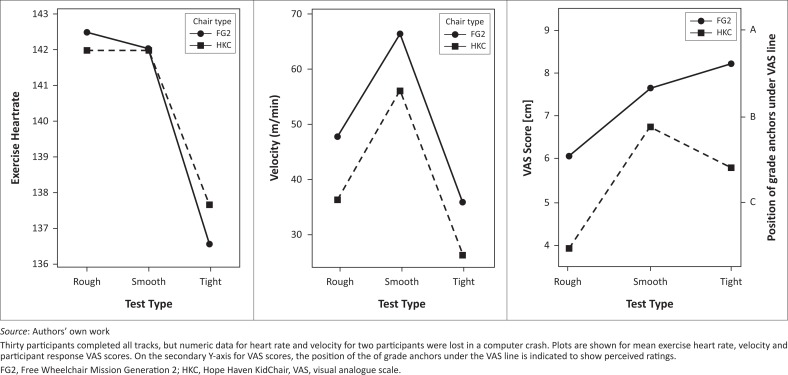
The analysis of variance interaction plots for the comparison between Free Wheelchair Mission Generation 2 and Hope Haven KidChair wheelchairs on rough, smooth and tight spaces rolling environments.

On rough, smooth and tight tracks, participants used both the HKC and FG2 wheelchairs. Anderson–Darling analysis indicated that distributions of data for velocity, mean exercise heart rate, and VAS participant response scores were statistically normal and suitable for parametric statistical analysis using ANOVA. Repeated measures ANOVA indicated significant differences in participant velocity (*F*(1,29) = 61.2, *p* < 0.001), and VAS scores (*F*(1,29) = 43.1, *p* < 0.001). Interaction plots for the ANOVA wheelchair factor showing mean values are given in Figure 3. Comparison of means indicated that the differences were driven by participants’ low velocity and low ratings for HKC (Figure 3). Although there were significant differences in exercise heart rate between tracks, there were no significant difference rates between wheelchairs. Of the participants, 40% provided qualitative comments. Comment topics as coded and counted across wheelchairs and tests are provided in Table 2.

**TABLE 2:** Coded and counted comments from the comparison between Free Wheelchair Mission Generation 2 and Hope Haven KidChair wheelchairs on rough, smooth and tight rolling environments (*n* = 30).

Type	Rough comments	Smooth comments	Tight comments
HKC	**Number and topic of negative comments appearing at least twice**		
	8 casters get stuck	3 wheels spin	5 difficult to turn
	7 wheels spin	3 slow and seems heavy	3 back uncomfortable
	5 wheels get stuck	2 arm hits tray knob	2 wheels too far back
	2 wheels too far back	2 takes too much energy	2 heavy and slow
	2 arm hits tray knob	2 casters turn & stick	
	**Number and topic of positive comments appearing at least twice**		
	5 seat comfortable	5 wheels roll well	3 easy to turn
	4 back wheels helpful	3 easy to turn going fast	3 comfortable
		2 brakes help with turning	
FG2	**Number and topic of negative comments appearing at least twice**		
	3 push rim bars hurt	3 push rim bars hurt hands	3 push rim bars hurt
	3 arms brush tire	3 difficult to turn going fast	3 arms brush tire
	4 casters got stuck		
	2 casters skid		
	**Number and topic of positive comments appearing at least twice**		
	3 wide wheels helpful	8 wide wheels helpful	7 casters helpful
	3 comfortable seat	6 casters helpful	6 push rims helpful

*Source*: Authors’ own work

HKC, Hope Haven KidChair; FG2, Free Wheelchair Mission Generation 2.

### Results for four-way comparison

**FIGURE 4:**
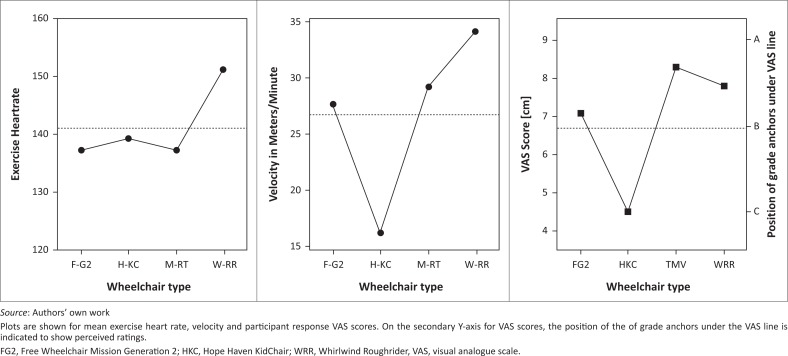
The analysis of variance main effects plots the comparison between Free Wheelchair Mission Generation 2, Hope Haven KidChair, Whirlwind Roughrider and MRT wheelchairs for the 12 participants able to complete the curb track in all four wheelchair types.

On the curb track, participants used all four wheelchair types. Chi-squared analysis indicated the proportion of participants able to complete the curb test did not differ between WRR, MRT and FG2 chairs, but was significantly lower for HKC chairs. Repeated measures ANOVA on data from the 12 participants capable of completing the test in all four wheelchair types indicated significant differences between wheelchairs for velocity (*F*(3,26) = 27.1, *p* < 0.001), VAS scores (*F*(3,26) = 5.80, *p* = 0.003) and exercise heart rate (*F*(3,26) = 63.26, *p* = 0.037). Main effects plots showing mean values are given in Figure 4. For all participants who completed a curb test in any of the wheelchairs, comments were coded and counted. Of the participants, 59% provided comments. Comment topics across wheelchairs are given in Table 3.

**TABLE 3:** Coded and counted comments from all runs in any wheelchair on the curb rolling environment.

HKC	FG2	WRR	MRT
**Number and topic of negative comments appearing more than twice**			
9 footplate hit ground	5 feels heavy	2 hands slip on push rims	5 feels heavy
3 feels heavy	3 push rim hurt hands	2 use a lot of energy	
2 hard to push	4 casters get stuck	2 feels heavy	
2 casters difficult	4 casters difficult		
2 wheels too far back			
2 back uncomfortable			
**Number and topic of positive comments appearing more than twice**			
3 push rims helpful	4 seat comfortable	9 wheels helpful	8 caster helpful
3 wheels helpful	3 wheels helpful	4 casters helpful	6 wheels helpful
2 seat comfortable		3 push rims helpful	5 comfortable seat
			2 push rims helpful

*Source*: Authors’ own work

HKC, Hope Haven KidChair; FG2, Free Wheelchair Mission Generation 2; WRR, Whirlwind Roughrider; MRT, Motivation Rough Terrain

## Discussion

Discriminatory validity of the AWMP was confirmed through ANOVA analysis which discerned significant differences between wheelchair types for velocity and participant response scores. The numbers and types of comment topics explained ANOVA results and added other qualitative information. Results from this study led to responsive design change confirming the value of the AWMP in providing comparative effectiveness data.

We had decided to use mean exercise heart rate in analysis instead of PCI for two reasons. We wanted to differentiate between two strategies of dealing with increased physiological difficulty, and reduce the effect of other factors that impact basal metabolic rate. Although mean exercise heart rate differentiated between rolling environments, we could see clearly that exercise heart rate, a measure of energy cost per minute, did not significantly differ between wheelchair types on the rough, smooth and tight tracks. If a wheelchair was more difficult to use, participants seemed to simply slow down. The only test for which ANOVA indicated a significant difference in exercise heart rate was the curb track for the 12 strong and skilled participants able to complete this test in all four chair types. This significant difference was because of higher mean heart rate in the WRR chair than in the other types. However, the significantly higher heart rate in WRR did not seem to impact perceived difficulty much, because visual analogue scores for WRR were not significantly different from those of the MRT wheelchair (Figure 2), and score patterns paralleled velocity more closely than heart rate. This seems to indicate that participants’ perception of difficulty was more closely related to velocity than heart rate. It would seem that by dropping the use of heart rate monitors from the protocol, we could simplify the AWMP considerably without much loss of discriminatory validity.

### Qualitative results

Open-ended qualitative conventional analysis methods resulted in the ability to elucidate explanatory topic categories. The relatively young age of the participants, with a mean age of 13 years, may have been a factor in the fact that fewer than 50% of the completed questions included explanatory comments. There was also no attempt to require that a comment be provided. In future studies, there could be more encouragement to provide explanatory comments. That said, the comments which were provided were helpful in understanding factors behind objective and subjective quantitative data. On the curb track, nine comments regarding the HKC indicated the footplate support framework hit the ground on descent from a curb unless the user was in a wheelie position. The slower velocities and lower completion rates likely reflect the greater difficulty of getting into a wheelie position before descending, a step unnecessary in the other wheelchair types – a finding which encouraged the development of the Bee line wheelchairs. Qualitative comments also resulted in responsive change in design by Free Wheelchair Mission. Participants who commented seemed to mention anything about the wheelchair that bothered them as they moved through the different rolling environments. This may have been something that did not slow them down. Although participants’ velocity was higher in FG2 than in HKC, many comments mentioned about the difficulty with the push rims on the FG2 chairs. When counted across all tracks, 12 comments mentioned discomfort from the bars which held push rims to wheel, and six comments mentioned push rims that caused forearms to brush the tire. Free Wheelchair Mission has responded with push rim modifications intended to address both issues.

For tests focused on the assessment of an individual’s mobility, four rolling environments may not be needed. Because of the very high individual variation in capability, a shorter test in one rolling environment such as the Wheelchair Propulsion Test differentiates between individuals (Askari et al. 2013). However, the goal of the AWMP is to assess the comparative effectiveness of mobility facilitated by different wheelchair designs. For that purpose, the four rolling environments were needed because design does not interact with each environment in the same way and there are multiple commonly encountered rolling environments.

Although the focus of this article is on the discriminatory ability of the AWMP rather than a direct comparison of the wheelchairs, a brief discussion of the factors which may be behind the evident differences may be of interest. Because the height of an obstacle that stops a wheel rolling is proportional to wheel diameter, and rolling resistance is inversely proportional to wheel size, one might have expected that the MRT chair with the largest wheels and caster would have done better than the other wheelchairs (Mason et al. 2012). However, centre of gravity relative to the axle position is also known to have an impact with a centre of gravity closer to the axle resulting in a lower rolling resistance partly because it offloads the smaller front caster (Lemaire et al. 1991). HKC has an anterior centre of gravity in comparison with the other three wheelchairs. It is interesting that results for velocity and participant response seemed to reflect this difference in centre of gravity more than the difference in wheel or caster size. However, HKC is the only chair of the four which includes options for head, trunk and hip supports; therefore, although it has often been distributed to active users at our study site, this may not be its ideal population. That said, the increase in rolling resistance is also present for pushers of wheelchairs, a factor that is of greater importance in low-resource areas with very little access to power wheelchairs (Sasaki & Rispin 2016).

The repeated measures protocol was key to enabling discriminatory validity in spite of wide variation among participants. The impact of differences because of disability characteristics, strength, gender, general outlook on life and age are minimised in a within subjects repeated measures protocol because each participant is only compared with themselves. For example, on the rough surface track, velocity among participants varied from 20 m/min to 70 m/min. The fastest participants travelled faster than the slowest participants in all chairs. However, repeated measures analysis could discern that there was a consistent pattern in which most participants travelled more slowly when in one wheelchair type. Data collection protocols that produced continuous statistically normal data was also important to discriminatory validity because it enabled ANOVA comparisons across wheelchairs and rolling environments. For example, ANOVA analysis enabled the communication of clear objective results showing that velocity and VAS responses were lower for HKC than the other wheelchair types (Figure 2). This was a key factor in Hope Haven’s exploration of other wheelchair design options. This study was completed in 2014. Hope Haven deployed the three wheeled ‘Bee-line’ wheelchairs in 2016 with the intention of facilitating better mobility in all commonly encountered rolling environments.

## Limitations and future work

Results of this study are specific to the conditions at our study site. Although it has much in common with other sites in low-resource areas, it is of course unique as are all locations. Tracks would not be identical at other study sites, and data from other study sites could not be directly compared with this study. For example, a rough ground track on an unpaved road at one location would not be exactly like a rough ground track at a different location. A low curb which is available and often encountered at one location might be 8 cm tall, whereas a curb at another location might be 10 cm tall. In studies carried out in North America, standardised rough surfaces have been developed and used (Sasaki & Rispin 2016). However, these standardised rough surfaces are large and not easily transported to field locations in low-resource areas. In addition, they would not perfectly model conditions encountered by wheelchair users. With the AWMP, study design, each participant is compared with themselves in their community location. AWMP could be used in many locations as long as there was no intention of considering the data exactly equivalent to data collected at another location. This flexibility is necessary in real-world research and is of key importance in studies carried out in low-resource areas. The use of letter grades as anchors for the VAS tends to standardise subject response and provide an understandable value to participants, researchers and manufactures (Funk et al. 2016; Rispin et al. 2017). However, the letter grades would need to be modified by whatever grading scale is in use in the culture in which a study is carried out.

Study wheelchairs were in the configuration most often utilised at our study site for those who can self-propel strongly. A broader comparison would have been provided if all four wheelchair types could have been included on all tracks, but this was not possible because of time constraints. Both the MRT and WRR chairs have the ability to be adjusted into less stable configurations with the centre of gravity closer to the rear axle, which, for skilled users, is thought to provide enhanced mobility (Motivation 2011; Whirlwind). Most wheelchairs at our study location had not been changed into this less stable but more energy efficient configuration. Therefore, we set the study WRR and MRT wheelchairs to a more stable configuration. Configurations of the same wheelchairs may routinely be set up differently at other study locations. However, additional studies at other locations are needed.

Results are specific to our study population of school age participants able to self-propel on rough surfaces. This population is not typical of the global population of wheelchair users, especially because obesity and age-related disability is becoming more prevalent in low-resource areas (WHO 2015). Validation is needed for the AWMP for other populations and cultures. However, it does seem likely that a wheelchair which is challenging for strong adolescent users will be even more challenging for older and more disabled users.

Wheelchair users were selected based on their identification by local therapists as having size and disability characteristics appropriate for the study wheelchairs. However, wheelchairs were not finely adjusted to each participant. Wheelchairs were of course not identical, and would have fit different participants somewhat differently. For each individual, this would impact results because appropriate seating is known to impact mobility (Borg, Larsson & Östergren 2011a). In this study, the sample size of 30 participants and the repeated measures format would have reduced the impact of the individual variation, unless the seating system of a wheelchair type was less suitable for most participants. In that case, the results were still likely of interest because the population of users with the types of wheelchairs in our study at our study location had the characteristics of our participant population. Results can be generalised only to the extent that other study sites may resemble our study site.

Wheelchair or user centre of gravity was not measured for each wheelchair and would have shifted somewhat for users of differing individual morphologies and resultant personal centres of gravity. However, body structure of individuals that resulted in a shifted centre of gravity would have remained the same for that individual in all chairs, so the within subjects protocol should have minimised the impact on comparative results.

All wheelchairs except HKC had inflatable tires. Before each data collection session, tire pressure was informally checked by pinching the tire, but was not formally checked using a tire gauge. A tire gradually losing air may not have been noticed and could have impacted results. In future studies, it would be wise to formally check tire pressure before each run.

The weather and other differences between days could have impacted our data. We chose to do each chair type on a different day because we felt that the fatigue which would result from attempting to do multiple wheelchairs on one day would have had a greater impact than any difference because of variations between days.

Accommodation to unfamiliar wheelchair types may also have impacted our data. Participants utilised multiple wheelchair types that included wheelchair types they had never used. A short accommodation period was included in the protocol; however, accommodation may take longer than a few minutes. If accommodation had affected results, FG2 results in this study could have been negatively impacted. A portion of participants were long-term users of each of the types of study wheelchairs. More participants were HKC users than any other chair, and yet all other study chairs outperformed the HKC chair. FG2 had the fewest long-term users.

### Future work

The use of research grade heart rate monitor and the ability to download data to a computer to calculate mean exercise heart rate could make the AWMP impractical at many locations. Because heart rate rarely differentiated between wheelchair types, in future studies, the use of exercise heart rate could be eliminated to make AWMP more broadly useful in low-resource areas. Modifications to the duration of the timed tests are also planned. Longer tests enable more sampling time for velocity; however, the added time can tire participants and challenge schedules (Kosak & Smith 2005). Studies indicate that for participants in self-propelling wheelchairs, the aerobic threshold is often considered to be reached 2 min from the initiation of exercise (Coutinho et al. 2014; Neto et al. 2014; Rispin & Wee 2015). Often, the impact of difficulty in propulsion may not be fully felt until a participant has passed this aerobic threshold. Four-minute tests would still include 2 min after this typical aerobic threshold, and should enable sufficient length of test for high discriminatory ability. The use of 4 min timed tracks on all rolling environments would also simplify data collection and analysis. This would allow a standard time across all tests, and would reduce the total time constraint on participants and researchers. Further testing is planned to confirm the benefit of the above changes.

Although face validity for this protocol is high, formal reliability testing and validity testing had not been performed at the time of this study. Test retest reliability and construct validity testing has now been carried out for the updated AWMP and results are presented in a companion study in this journal.

## Conclusion

The good discriminatory validity of the AWMP enables its use in comparative effectiveness studies that can provide much needed feedback enabling wheelchair manufacturers to optimise wheelchair design. Organisations that design and manufacture wheelchairs intended for use in low-resource settings are almost always not-for-profit and have a strong commitment to meeting the overwhelming global need for wheelchairs. Designs that hinder users’ abilities to roll forward on commonly encountered rolling environments limit the positive impact of wheelchair provision. The AWMP can be used in comparative effectiveness studies for other wheelchair types to inform beneficial design change. If AWMP studies are performed with locally available wheelchairs in locations where wheelchair users live, results could enable informed choices for wheelchair provision. Other stakeholders such as granting or charitable agencies providing funding could also benefit from comparative effectiveness data to inform wheelchair selection choices for different locations.
